# Supplementary material for: An outbreak of pulmonary tuberculosis and a follow-up investigation of latent tuberculosis in a high school in an eastern city in China, 2016–2019
Source: PLoS One. 2021 Feb 24;16(2):e0247564. doi: 10.1371/journal.pone.0247564 (PMC7904191; doi:10.1371/journal.pone.0247564)
Supplement: S3 File — (DOC) [file pone.0247564.s003.doc]

**Questionnaire for Patients with TB infection**

Hello! I’m X of Shaoxing CDC. TB cases were seen in Y school recently and we have found that you are probably infected with TB. We would inquire of you relevant information, including issues related to incurrence, exposure, diagnosis and treatment. These questions would not conflict with your interests. Your response and answer would contribute to the control of TB outbreak in Y school. We are looking forward to your support and cooperation.

You take part in this investigation voluntarily. You may withdraw from the investigation at any time without giving a reason and without your withdrawal affecting your usual care and treatment.

If you agree to participate in this investigation, please sign your name below. If you are less than 18 years old, your parent or other legal guardian shall sign for you if you agree to participate.

Thank you for your participation and support!

**Signature of participant: Date:**  _____

**Category of diagnosis**:

□ Laboratory confirmed □ Clinical case □ Suspected case

Case number □□□□

**1. General information**

1.1 Name：

1.2 ID number: _______________________

Current address：______________________________________

1.3 Gender： □Male □Female

1.4 Birth date： ____ or Age years

1.5 Category of occupation：□ Children □Students in campus

□Teachers in campus □Health care worker □Catering service

□Commercial/service workers □Industrial workers with non-agriculture household registration □Farmers who engage in non-agricultural labor □Agricultural workers (involved in forestry, animal husbandry or fishery) □Retired people □Other

1.6 Education background：□Primary school □Junior high school or equivalent education □Senior high school or equivalent education □Junior college or above □Illiterate or semiliterate □Unknown

1.7 Telephone number:

1.8 For students only, Grade ___ Class_______，the number of the students in the class

1.9 Dormitory：Building Room_____，the number of the roommates

Area of the room(sqm) ；Area of the window(sqm) ；

Ventilation: □Not done □Done by un-regularly □Done by daily

Air conditioner usage hours/day

Sanitation：□Good □Average □Bad

**2. History of disease and contact**

2.1 Previous TB disease history：□Yes(onset date ____)

□No

2.2 Previous disease history □Chronic hepatic disease □Chronic renal disease □Diabetes

2.3 Smoking history：□Current smoker □Quitted □Never

2.4 Based on your knowledge, whether there are TB cases in below groups？

□Family members □Classmates □Roommates

If yes, whether there were any contact with any cases？□Yes □No

□Unknown

**3．Nutrition and other health conditions**

3.1 Nutrition status：□Good □Average □Bad

3.2 Sleeping status：□Good □Average □Bad

3.3 Stress from learning, work and life：□Huge □Average □Minor

**4. Onset and hospital visit**

4.1 Presented with symptoms：□Yes □No

If yes，onset date： _______

4.2 Initial symptom(Tick with √)：□Cough □Expectorate sputum

□Hemoptysis or sputum with blood □Chest distress and short breath

□Low fever □Night sweat □Weakness □Anorexia □Other

4.3 Severity of initial symptom judged by self-perception:

□Mild □Average □Severe

4.4 Hospital visit

| Sequence | Visit date | Primary cause for visit | Hospital being visited | Diagnosis | Treatment |
| --- | --- | --- | --- | --- | --- |
| 1(Initial visit ) |  |  |  |  |  |
| 2 |  |  |  |  |  |
| 3 |  |  |  |  |  |
| …… |  |  |  |  |  |

**5．Diagnosis and treatment**

5.1 Diagnosed date： _________

5.2 Diagnosed institution：

5.3 Whether Suspension of schooling/working for treatment after being diagnosed: □Yes □No

If yes, the date for suspension: _____

**6. Contact history and information about close contacts**

6.1 Are there any TB cases among your neighbouring population such as family members, relatives, neighbors, colleagues, classmates and friends etc? If yes, who is TB case and what’s the relationship with you? For instance, same row in the class, eat together, live together or hang around together?

| Name of the case | Gender | Age | Relationship with you | Onset date | The way of contact | Time period of contact | Location of contact | Current address | Telephone number |
| --- | --- | --- | --- | --- | --- | --- | --- | --- | --- |
|  |  |  |  |  |  |  |  |  |  |
|  |  |  |  |  |  |  |  |  |  |
| …… |  |  |  |  |  |  |  |  |  |

6.2 Who(such as family members, relatives, neighbors, colleagues, classmates and friends etc.) are in close relationship with you regarding to same row in the class, eat together, live together or hang around together?

| Name of the contact | Gender | Age | Relationship with you | The way of contact | Time period of contact | Location of contact | Current address | Telephone number |
| --- | --- | --- | --- | --- | --- | --- | --- | --- |
|  |  |  |  |  |  |  |  |  |
|  |  |  |  |  |  |  |  |  |
| …… |  |  |  |  |  |  |  |  |

6.3 Draw the distribution map of the classroom/workshop

6.4 Draw the distribution map of the dormitory

**7．Clinical records (Acquired from specialized TB institution or designated hospital directly)**

7.1 The way to identify the patient: □Hospital visit due to presenting symptoms □Transfer treatment □Follow up □Recommended due to suspected TB symptoms □Examination for the contacts □Physical examination(Mass screening) □Other

7.2 Tuberculin(Mantoux) skin test (mm)： mm； Date Tuberculin skin test(TST) placed： ______

7.3 Chest radiograph and other chest imaging study:

Left side□Abnormal, consistent with TB(If yes，please indicate，upper、middle、lower) □Normal

Right side □Abnormal, consistent with TB (If yes，please indicate，upper、middle、lower) □Normal

Evidence of a cavity □Yes □No

Evidence of miliary TB □Yes □No

7.4 Laboratory Examination for Sputum

Sputum Smear □Positive □Negative □Not Done

Sputum Culture □Positive □Negative □Specimen Contaminated □Not Done

Initial identification for the isolate：□ mycobacterium tuberculosis □nontuberculous mycobacteria,NTM  □Other

Drug Susceptibility Testing

H □Resistant □Sensitive □Specimen Contaminated □Not Done

R □Resistant □Sensitive □Specimen Contaminated □Not Done

S □Resistant □Sensitive □Specimen Contaminated □Not Done

E □Resistant □Sensitive □Specimen Contaminated □Not Done

7.5 Diagnosis：

7.6 Category of diagnosis：□TypeⅠ□TypeⅡ□TypeⅢ□TypeⅣ □TypeⅤ

7.7 Date of registration： _____

7.8 Category for the patient: □New □Recurrence □ Reverse back

□Initial failure □Other

7.9 Date of therapy started ： ___

7.10 Drug regimen：

7.11 The way to receive the treatment：□Suspension of schooling/working and hospitalized □Suspension of schooling/working and stay at home

□Continue school/work □Back to native residence area

Signature of investigator ：

Date：
