# Supplementary material for: An outbreak of pulmonary tuberculosis and a follow-up investigation of latent tuberculosis in a high school in an eastern city in China, 2016–2019
Source: PLoS One. 2021 Feb 24;16(2):e0247564. doi: 10.1371/journal.pone.0247564 (PMC7904191; doi:10.1371/journal.pone.0247564)
Supplement: S4 File — (DOC) [file pone.0247564.s004.doc]

肺结核病例个案调查表

诊断分类: □ 实验室诊断 □ 临床诊断 □ 疑似 病例编码 □□□□

1.一般情况

1.1姓名：

1.2身份证号码: _______________________

现住址：______________________________________

1.3性别： □男 □女

1.4出生日期： 年 月 日 或年龄 岁

1.5职业：□ 幼托儿童 □学生 □教师 □医务人员 □餐饮业 □商业服务 □工人 □民工 □农民 □离退人员 其他

1.6 文化程度：□学龄前儿童 □小学 □初中 □高中或中专 □大学及以上

□文盲 □不详

1.7联系电话:

1.8如是学生,_________________________年级 班，班级人数 人

1.9寝室： 幢 室，同室居住人数 人

宿舍面积(平方米) ；窗户面积(平方米) ；

通风情况: □不开窗通风 □不定时开窗通风 □每日开窗通风

空调 小时/天

宿舍环境卫生：□好 □一般 □差

2.既往病史和接触史

2.1既往结核病史：□有(患病时间 年 月) □无

2.2 既往疾病史 □慢性肝病史 □慢性肾病史 □糖尿病史

2.3 吸烟史：□现在吸 □以前吸 □从不吸

2.4 据你所知，发病前，以下人群中是否有结核病患者？

□家庭人员 □同班级人员 □同宿舍人员

若有，是否与患者密切接触？□是 □否 □不知道

3．营养和其他健康状况

3.1 营养状况：□好 □一般 □差

3.2 睡眠状况：□好 □一般 □差

3.3 学习、工作和生活压力：□大 □一般 □小

4.发病和就诊情况

4.1是否有症状：□有 □无

若有，首次发病日期(或症状出现日期)： 年 月 日

4.2首次发病出现症状(打√)：□咳嗽 □咯痰 □咯血或血痰 □胸闷及气短 □低热 □盗汗 □乏力 □食欲减退 □其他

4.3首发症状自我感觉的严重程度: □轻 □中 □重

4.4就医过程

| 就诊序次次 | 就诊日期(年月日) | 就诊主要原因 | 就诊单位 | 诊断结果 | 治疗情况 |
| --- | --- | --- | --- | --- | --- |
| 1(初诊) |  |  |  |  |  |
| 2 |  |  |  |  |  |
| 3 |  |  |  |  |  |
| …… |  |  |  |  |  |

5．确诊和治疗情况

5.1确诊日期： 年 月 日

5.2确诊单位：

5.4确诊后是否休学/休工治疗: □是 □否

如是，休学/休工开始日期: 年 月 日

6.详细接触史与密切接触者情况

6.1你周围人群(如家人、亲戚、邻居、同事、同学、朋友)中有无结核病患者?如有为谁？与你的关系如何(如同座、同吃、同住、同玩)？

| 患者  姓名 | 性别 | 年龄 | 与你  关系 | 患病  日期 | 接触  方式 | 接触  时间 | 接触  地点 | 现住址 | 联系  方式 |
| --- | --- | --- | --- | --- | --- | --- | --- | --- | --- |
|  |  |  |  |  |  |  |  |  |  |
|  |  |  |  |  |  |  |  |  |  |
| …… |  |  |  |  |  |  |  |  |  |

6.2你周围人群(如家人、亲戚、邻居、同事、同学)与你的关系(如同座、同吃、同住、同玩)比较密切的有哪些?

| 接触者  姓名 | 性别 | 年龄 | 与你  关系 | 接触  方式 | 接触  时间 | 接触  地点 | 现住址 | 联系  方式 |
| --- | --- | --- | --- | --- | --- | --- | --- | --- |
|  |  |  |  |  |  |  |  |  |
|  |  |  |  |  |  |  |  |  |
| …… |  |  |  |  |  |  |  |  |

6.3绘出教室/车间分布图

6.4绘出宿舍分布图

7．患者的临床诊治资料(从结防机构或定点医院的病案资料直接获取)

7.1病人发现方式: □因症就诊 □转诊 □追踪 □因症推荐 □接触者检查 □健康检查(集中筛查) □其他

7.2 结素试验(PPD)结果(mm)： mm； 试验日期： 年 月 日

7.3 X线胸片检查异常情况:

左 □有(若有，请表明，上、中、下) □无

右 □有(若有，请表明，上、中、下) □无

空洞 □有 □无；

粟粒 □有 □无

7.4痰液实验室检查结果：

痰涂片结果 □涂阳 □涂阴 □未查

培养结果 □涂阳 □涂阴 □污染 □未查

初步菌种鉴定结果：□结核分枝杆菌 □非结核分枝杆菌 □其他

药敏结果 H □耐药 □敏感 □污染 □未做

R □耐药 □敏感 □污染 □未做

S □耐药 □敏感 □污染 □未做

E □耐药 □敏感 □污染 □未做

7.5诊断结果：

7.6诊断分型：□Ⅰ型 □ Ⅱ型 □ Ⅲ型 □Ⅳ型 □Ⅴ型

7.7登记日期： 年 月 日

7.8登记分类: □新患者 □ 复发 □ 返回 □初治失败 □其他

7.9开始治疗日期： 年 月 日

7.10治疗方案：

7.11治疗管理方式：□休学/休工住院治疗 □休学/休工本地居家治疗

□未休学/休工在校治疗 □回外地原籍治疗

调查单位： 调查者 ： 调查时间： 年 月 日
