# Supplementary material for: An outbreak of pulmonary tuberculosis and a follow-up investigation of latent tuberculosis in a high school in an eastern city in China, 2016–2019
Source: PLoS One. 2021 Feb 24;16(2):e0247564. doi: 10.1371/journal.pone.0247564 (PMC7904191; doi:10.1371/journal.pone.0247564)
Supplement: Attachment — Submitted filename: Response letter20210201.docx [file pone.0247564.s006.docx]

Dear Editor,

Thank you for the reviewers’ comments on our manuscript, "An outbreak of pulmonary tuberculosis and follow-up of latent tuberculosis infection in a high school: China, 2016-2019" (PONE-D-20-26099) and the opportunity to address the reviewer’s comment and revise our manuscript. Reviewer 4 answered “yes” to all of the questions, with the exception of Question 6, in which he or she requested further explanation. This comment is constructive and valuable, and helped us to clarify a limitation of our case definition. In this letter, we describe our response to the comment on Question 6, with the reviewer’s comment in bold font and our response in normal font. The manuscript is provided in track-changes as requested.

Sincerely,

Yingxin Pei

**Reviewers' comments:**

**Reviewer's Responses to Questions**

**6. Review Comments to the Author
Reviewer #4:**

**There still is a problem about the definition on latent TB infection.  Based on guideline, latent TB infection is that TSTs is over 10 mm.  For preventive therapy, the cut-off point is recommended as TSTs 15 mm for students. The author should clarify it.**

Authors’ Response:

The reviewer makes a good point. We defined latent TB infection (LTBI) as a TST with induration ≥15mm in diameter in a student or teacher of the high school. As the reviewer points out, this cut-off point is the value for initiating prophylaxis in our national TB guidelines. Our use of 15 mm for the definition of LTBI is more restrictive than a 10 mm cut-off, and will identify fewer individuals in need of prophylaxis. Since we used national guidelines for definitions, this is a limitation of our study. We now recognize this as a limitation in the manuscript, and we state in the limitations section, “Third, as per national guidelines for prophylaxis, we defined latent TB infection (LTBI) as TST induration ≥15mm in diameter. This cut-off is more conservative than a ≥10mm cut-off, and will result in fewer LTBI cases being shown to progress to TB than would have been shown had a 10 mm cut-off been used.”
